# Serum long noncoding RNA H19/micro RNA-675-5p axis as a probable diagnostic biomarker in inflammatory bowel disease

**DOI:** 10.1007/s11033-023-08777-8

**Published:** 2023-09-16

**Authors:** Olfat G. Shaker, Aya Safa, Ahmed Khairy, Naglaa F. Abozeid

**Affiliations:** 1 Medical Biochemistry and Molecular Biology, Faculty of Medicine, Cairo University, Giza, Egypt; 2 Endemic Medicine, Faculty of Medicine, Cairo University, Giza, Egypt

**Keywords:** Inflammatory bowel disease, LncRNA, H19, miRNA-675

## Abstract

**Background:**

A significant body of research strengthens the starring role of long non-coding RNAs (lncRNAs) and microRNAs (miRNAs) in the pathogenesis of inflammatory bowel disease (IBD). Here, we investigated the diagnostic utility of lncRNA H19 and miRNA-675-5p in IBD.

**Methods:**

This study included 97 participants, thirty-five ulcerative colitis patients, thirty-two Crohn’s disease patients, and thirty IBD-free controls. History, staging, laboratory investigations, and colonoscopy were performed. Also, quantitative real-time PCR (qPCR) for revealing of lncRNA H19 and miRNA-675-5p was done.

**Results:**

The estimated serum levels for H19 and miRNA-675-5p in the UC and CD groups in comparison to the control group showed a high statistical difference (P = 0.0001 for each parameter). Based upon the severity of UC patients, both biomarkers showed significantly higher values between remission and moderate cases, with p-values 0.022 and 0.02, respectively. Meanwhile, in CD patients, both biomarkers revealed no statistical significance between remission and any active stage of the disease. Additionally, ROC analysis revealed that H19 could discriminate between UC and control subjects with 94.3% sensitivity and 90.0% specificity, and with 87.5% sensitivity, and 88.5% specificity in the CD group. Furthermore, miR-675-5p was able to discriminate between UC and control subjects with 85.7% sensitivity and 97.3% specificity and with 88.4% sensitivity, 95.2% specificity in the CD group. Logistic regression found a significant predictive utility of using miR-675-5p and H19 in IBD.

**Conclusion:**

H19 and miRNA-675-5p can be used as diagnostic biomarkers in IBD, with superiority in UC patients with moderate activity.

## Introduction

Inflammatory bowel disease (IBD) principally comprises ulcerative colitis (UC) and Crohn’s disease (CD), two phenotypes of the gastrointestinal tract (GIT) that share the chronicity of inflammation but differ in symptoms, disease location, and histopathological characteristics [1]. The incidence of IBD rises globally, with suspected attribution in part to the westernization of lifestyles [2]. The pathogenesis of IBD is not fully known; however, evidence suggests it develops via the convergence of immune factors, environmental factors, gut microbiota, and genetic susceptibility [3]. The disease course has an oscillating evolution between relapse and remission, which requires effective monitoring and evaluation [4]. Endoscopy is widely accepted as the main method for diagnosis [5], and currently, only C-reactive protein (CRP) and faecal calprotectin are used as reliable markers in IBD management [6]. However, more non-invasive biomarkers are admired, especially for patients with mild symptoms, in order not to burden their quality of life.

In recent literature, new insights have been associating IBD with noncoding RNAs, as they have arisen as vital regulators of gene expression at both the transcriptional and post-transcriptional levels [7]. Some have a protective role by maintaining gut microbiota homeostasis and regulating intestinal inflammation [8], but most of them are implicated in its pathogenesis through alterations in autophagy, intestinal barrier, and immune homeostasis [9]. They include long noncoding RNAs (lncRNAs) [7, 10] and microRNAs (miRs or miRNAs) [11, 12]. Some lncRNAs may contain miRNAs in their sequence that can be released by splicing, e.g., lncRNA H19 includes miRNA-675-3p and 675-5p [13].

The telomeric lncRNA H19 is a maternally imprinted gene located on chromosome 11. It is expressed at a high rate during fetal development but is downregulated postnatally [14]. It was linked to many cancers as an oncogene [15]. H19 and its intragenic miRNA-675 have been, together, studied in a plethora of cancers: colorectal [16], breast [17], glioma [18], rhabdomyosarcoma [19], gastric cancer [20], and liver cancer [21].

The expression of lncRNAs has been found to be abnormal in inflammatory diseases [22]. Regarding IBD, H19 levels increase markedly in the inflamed intestinal mucosa, predominantly due to an increase in interleukin 22 [23]. It may cause dysfunction of the epithelial barrier by suppressing autophagy [24]. MiRNA-675 is encompassed in miRNAs that weaken the intestinal barrier through destabilizing Cadherin E and zonula occludin 1 (ZO-1) mRNAs that are translated into tight junction proteins and adherens junction proteins [25].

In the current study, we aim to determine the diagnostic utility of lncRNAH19 and miR-675-5p as novel biomarkers in IBD.

## Subjects and methods

### Study population

This case-control study is a prospective basis of 97 Egyptian adult individuals, who were split into 3 groups. They were randomly chosen from the outpatients of the Tropical Medicine Department, Faculty of Medicine, Kasr Alayni Hospitals.

1. UC Group: 35 UC patients (14 (40%) females and 21 (60%) males) with a mean age of 32.06 ± 1.88 years.
2. CD Group: 32 patients with Crohn’s disease (11 (34.4%) females and 21 (65.6%) males) with a mean age of 33.84±2.10 years.
3. Control Group: 30 IBD-free patients (a colonoscopic and histopathological diagnosis; their symptoms are proven not to be caused by IBD) (15 (50%) females and 15 (50%) males) with a mean age of 32.07 ± 0.85 years.

#### Inclusion criteria

A minimum age of 18 years with a proven diagnosis of IBD, based on clinical, laboratory, endoscopic, and histological examinations.

#### Exclusion criteria

Patients with other causes of chronic diarrhea, malignancy, concurrent auto-immune or endocrine disease, a history of ongoing infection, and patients who are pregnant, lactating, or using estrogens for any reason.

All the members of the study group were subjected to history taking, physical examination, blood sampling for RNA extraction (the serum was separated and stored at -80 °C), and laboratory investigations, namely complete blood count (CBC), C-reactive protein (CRP), erythrocyte sedimentation rate (ESR), and serum albumin. In addition, a terminal ileoscopy and biopsy were performed, and then the specimens were sent for histopathology for confirmation of the diagnosis.

Activity was assessed clinically according to Peyrin-Biroulet et al. [26], where:

- Crohn’s disease activity index (CDAI score) for CD defines remission in CD patients as a score below 150, 150–219 to be moderate, and > 450 to be severe.
- Mayo score for UC: where a Mayo score < 2 is considered to be in remission, 3–5 are considered mild, 6–10 are considered moderate, and 11–12 are considered severe UC.

### Chemicals and equipment

- For the relative quantitative measurement of lncRNA H19 and miRNA-675-5p, QIAzol lysis reagent was added to 200 µL serums, and the miRNeasy mini kit for purification of serum total RNA, the miScript II RT kit for reverse transcription (RT), and miScript SYBR Green PCR kit for qPCR (Qiagen, USA) were used in line with the manufacturer’s instructions. The primers used are Hsa-miR-675-5p, Cat No. MS00032109, and SNORD 68, Cat No. MS00033712 (due to the lack of an endogenous reference housekeeping gene of miRNA in the serum), H19, Cat No. LP 01147 A and GAPDH QT 300,079,247 (as the endogenous housekeeping gene). The sequence of the primer pairs used for the qPCR are shown in Table 1.
- Using the NanoDrop® (ND)-1000 spectrophotometer (NanoDrop Technologies, Inc., USA), RNA quantification and purity were evaluated.
- qPCR was programmed using the Rotor-gene thermocycler (Qiagen, USA).

**Table 1:** Primers sequence of the studied genes

Gene	Sequence of Primer from 5′- 3′ F: Forward primer, R: Reverse primer
**lncRNA H19**	F: GCACCTTGGACATCTGGAGT R: TTCTTTCCAGCCCTAGCTCA
**GAPDH**	F: TGAAGGTCGGAGTCAACGGATTTGGT R: CATGTGGGCCATGAGGTCCACCAC
**miRNA-675-5p**	F: TTGGTGGTGCGGAGAG-3′ R: AGTGCGTGTCGTGGAGTC-3′

### Calculation of results and statistical analysis

The expression levels of miR-675-5p and lnc H19 were evaluated using the ΔCt method, where the fold change in their expression levels was calculated by the Eq. 2^–ΔΔCt^. Data were analyzed using SPSS 17.0. For quantitative parametric data, arithmetic means were generated to measure central tendency, while the standard error was utilized as a measure of dispersion. The independent student t-test and the one-way ANOVA test, one for comparing two independent groups and the other for more than two independent groups, were utilized for the quantitative parametric data. Using Benferroni Post-Hoc, significance was tested. The Kruskal-Wallis and Mann-Whitney tests were employed for non-parametric data to compare more than two independent groups and to determine any statistical significance between the groups. Bivariate Pearson correlation analysis with a two-tailed test of significance was employed to measure the correlation between groups. With the ROC Curve (Receiver Operating Character), sensitivity and specificity tests were created. A P-value cutoff of 0.05 was used [27].

## Results

### Demographic characteristics and clinical data of the study groups

**Fig. 1:**
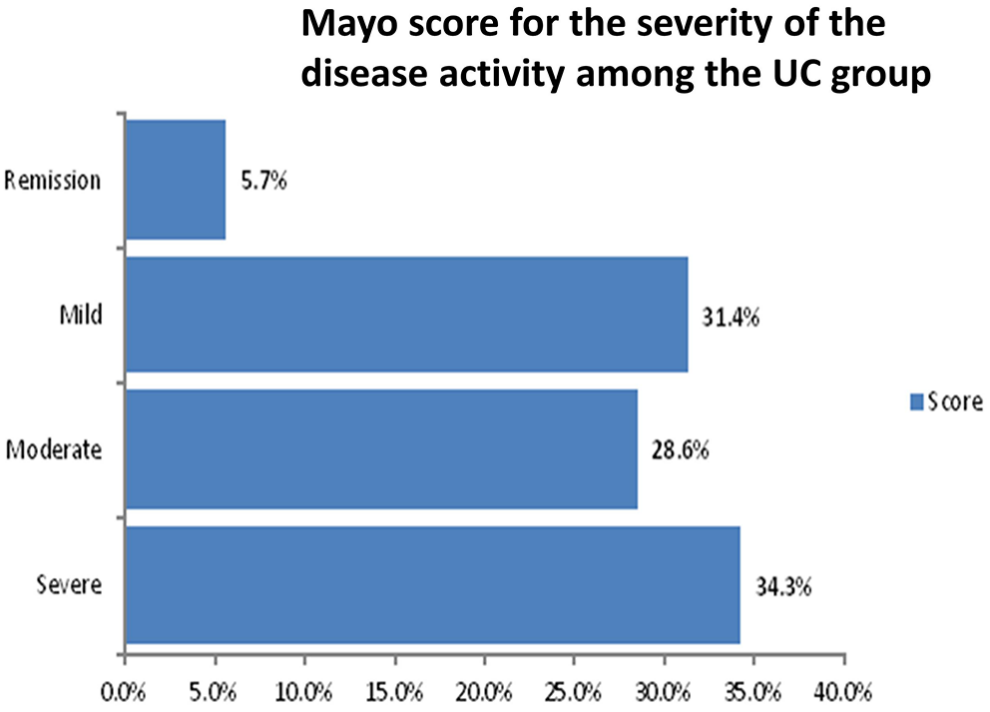
Mayo score for the severity of the disease activity among the UC group

**Fig. 2:**
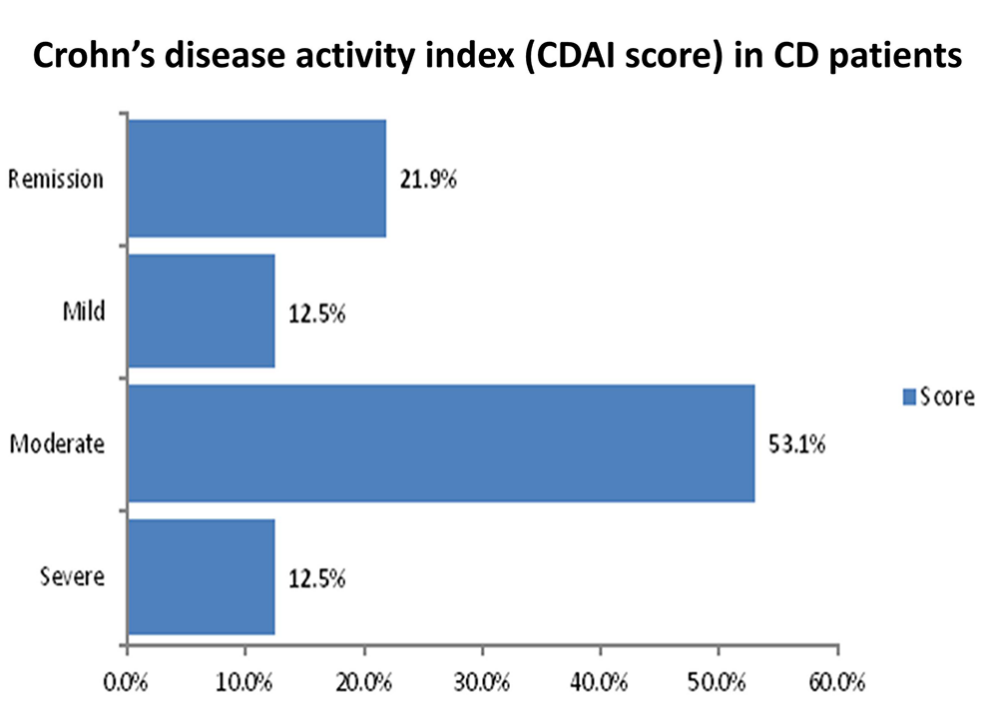
Crohn’s disease activity index (CDAI score) in CD patients

Matching the control group, most of the recruited patients were in their early thirties with a low percentage of concurrent chronic diseases. 75% of CD patients have experienced extra-intestinal manifestations. According to the severity of the disease, the majority of the UC patients (34.3%) showed severe activity using Mayo score, while most of the CD patients (53.12%) showed moderate activity using CDAI score. More details can be depicted in Figs. 1 and 2, and Table 2.

**Table 2:** Demographic data and clinical features for the study groups

Clinical Data	Control (n = 30)	UC (n = 35)	CD (n = 32)
Age (years)	32.07±0.85	32.06±1.88	33.84±2.10
Gender: Female Male	15 (50%) 15 (50%)	14 (40%) 21 (60%)	11 (34.4%) 21 (65.6%)
Smoker	2(6.67%)	8(22.9%)	11(34.3%)
Coffee consumption	6(20%)	6(17.1%)	11(34.3%)
Diabetes	0(0%)	4(11.4%)	1(3.1%)
Hypertensive	1(3.3%)	1(2.9%)	1(3.1%)
Duration (Months)	Not applicable	40.36±9.21	52.32±11.41
Extra-intestinal manifestations	Not applicable	14(40%)	24(75%)

### Laboratory investigations of the study groups

Both UC and CD patients showed significantly increased levels of inflammatory markers (CRP and ESR) compared to the control group. They also experienced a significant elevation in total leucocyte count (TLC), neutrophils and platelets, and decreased albumin levels. In addition, UC- but not CD- patients have a significant decrease in hemoglobin level compared to the control group, as shown in Table 3.

**Table 3:** Laboratory investigations of the study groups

Laboratory investigations	Control (n = 30)	UC (n = 35)	P-value (UC/ control)	CD (n = 32)	P-value (CD/ control)
**Hb** (g/dl)	12.48±0.24	11.23±0.33	**0.005**	12.07±0.35	0.34
**Htc** (%)	39.10±0.62	34.39±1.04	**0.029**	36.32±1.04	**0.028**
**TLC** (× 10^3^/mm^3^)	6.23±0.25	8.28±0.82	**0.030**	8.51±0.72	**0.005**
**Neutrophils** (× 10^3^/mm^3^)	48.72±1.14	60.77±2.82	**0.001**	60.33±2.23	**0.001**
**Platelets** (× 10^3^/mm^3^)	296.67±11.29	317.34±18.28	**0.042**	350.66±23.43	**0.001**
**CRP** (µg/ml)	1.99±0.22	17.48±3.53	**0.0001**	29.15±7.41	**0.001**
**ESR** (mm/h)	7.23±0.30	31.74±4.48	**0.0001**	34.53±5.02	**0.0001**
**Albumin** (g/dl)	4.58±0.049	3.66±0.14	**0.001**	3.59±0.12	**0.0001**

Data are shown as mean ±SE, One-way ANOVA is used for calculating the p-value. P values in bold are statistically significant (P < 0.05). Hb: hemoglobin, Htc: hematocrite, TLC: total leucocyte count, CRP: C-reactive protein, ESR: Erythrocyte sedimentation rate

### Serum levels for H19 and miRNA-675-5p in the study groups

**Fig. 3:**
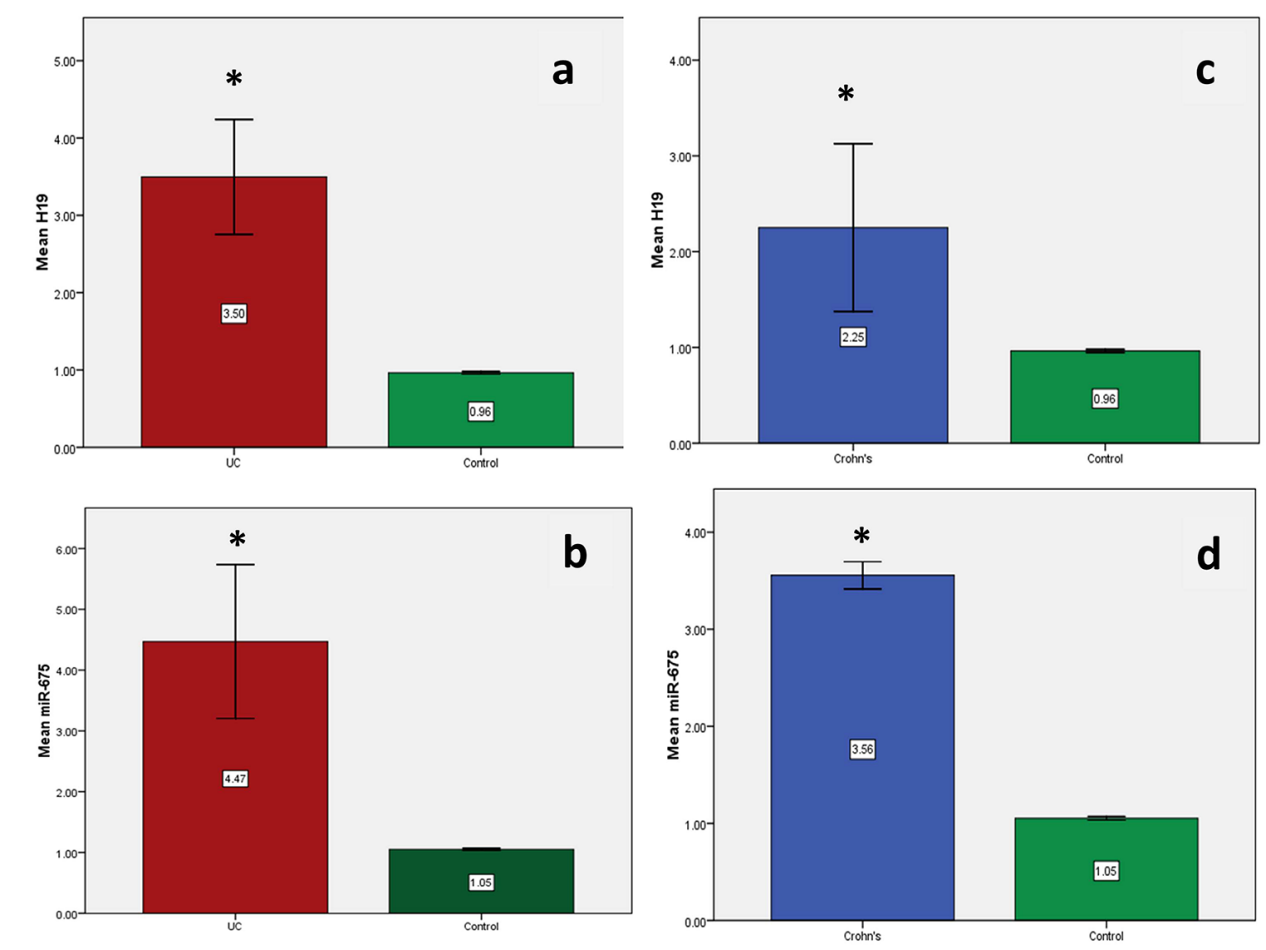
Mean serum levels for H19 and miRNA-675-5p using qPCR in the study groups. **a**) Serum H19 gene expression levels between UC group and control group. **b**) Serum miR-675 gene expression levels between UC group and control group. **c**) Serum H19 gene expression levels between CD group and control group. **d**) Serum miR-675 gene expression levels between CD group and control group. * = p value is significant (< 0.05) against the control group

The estimated mean serum levels for H19 and miRNA-675-5p in the UC and CD groups in comparison to the control group showed a high statistical difference (P = 0.0001 for each parameter), as shown in Fig. 3.

### Relationship between serum biomarkers miR-675-5p, lncH19 and the severity of the disease in the study groups

**Fig. 4:**
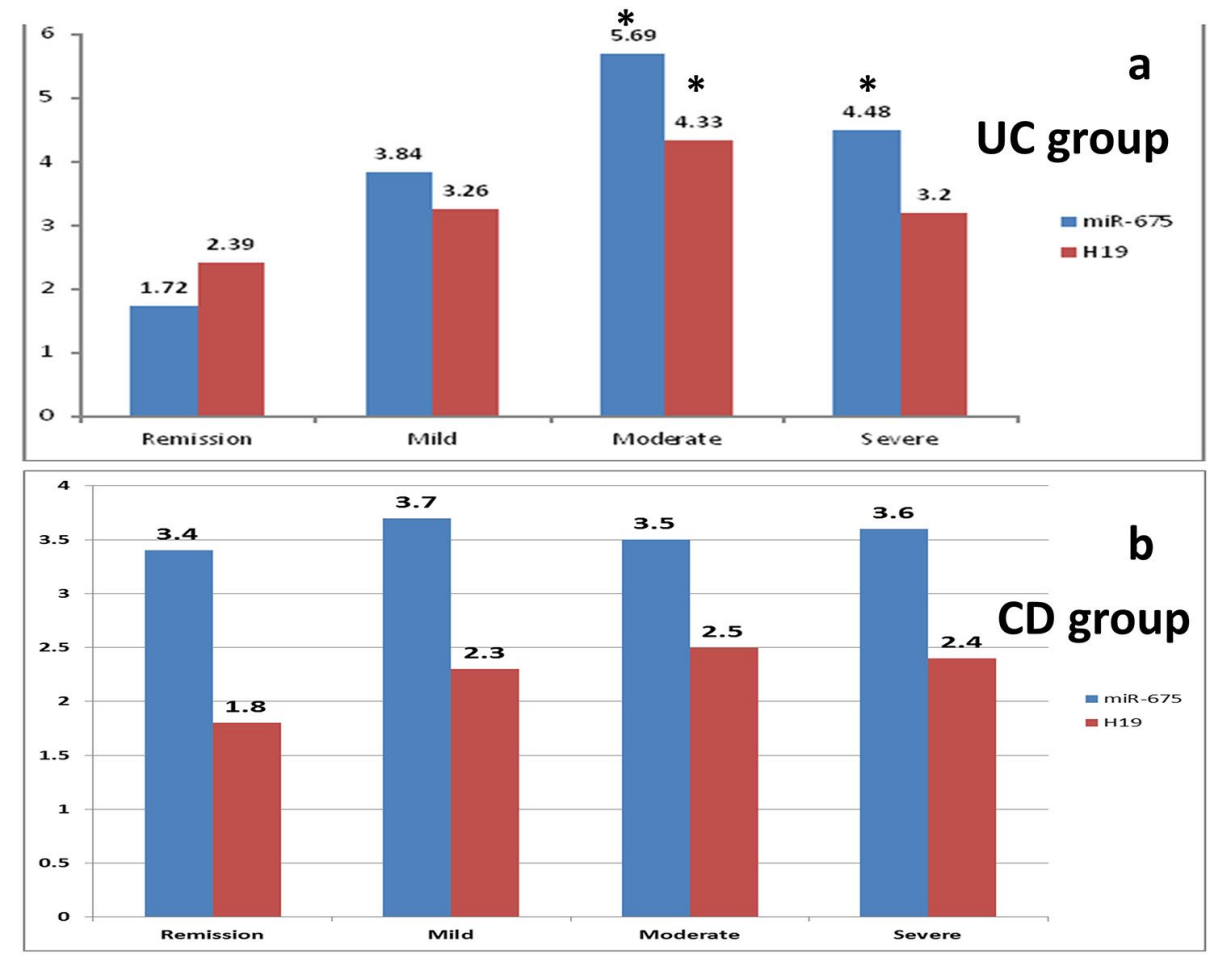
Relationship between serum biomarkers miR-675-5p, lncH19 and the severity of the disease in the study groups. **a**) Serum biomarkers gene expression levels in the UC group (severity is determined according to Mayo score**)**. **b**) Serum biomarkers gene expression levels in the CD group (severity is determined according to CDAI score). * = p value is significant (< 0.05) against the remission stage

Based upon the severity classification in UC patients, miR-675-5p showed significantly higher values between remission and both moderate and severe cases, with p-values 0.02, 0.037 respectively [Fig. 4]. Besides, H19 showed significantly higher values between remission and moderate cases only (p-value = 0.022). While in CD patients, both H19 and miRNA-675-5p showed fluctuating non-significant difference between remission and any active stage of the disease.

### ROC curve to evaluate the diagnostic and prognostic performances of miR-675-5p and lncH19 in the study groups

**Fig. 5:**
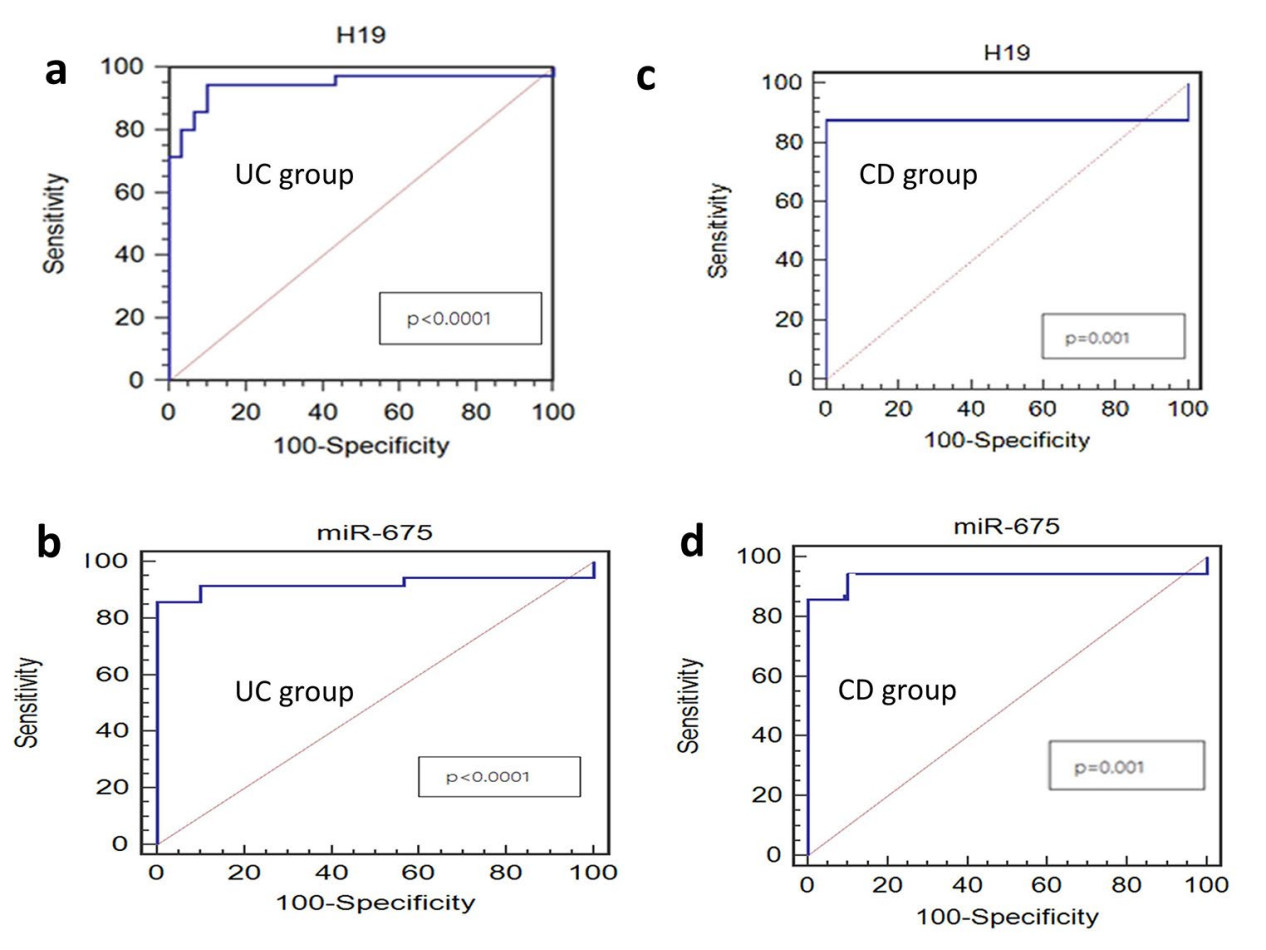
ROC curve for serum biomarkers in the study groups. **a**) H19 between UC group and control group. **b**) MiR-675-5p between UC group and control group. **c**) H19 between CD group and control group. **d**) MiR-675-5p between CD group and control group. P-value < 0.05 was considered as a cutoff value for significance

Using ROC curve analysis in UC patients, H19 was able to discriminate them from control subjects with a cut-off level of 3.53, 94.3% sensitivity, and 90.0% specificity (AUC, area under curve = 0.944, p-value < 0.0001) (Fig. 5a). MiR-675-5p showed a cut-off level of 4.56 with 85.7% sensitivity, and 97.3% specificity (AUC = 0.921, p-value < 0.0001) (Fig. 5b).

In CD patients, H19 has a cut-off level of 2.04, with 87.5% sensitivity and 88.5% specificity (AUC = 0.875, p-value = 0.001) (Fig. 5c). MiR-675-5p level has a cut-off of 3.65, with 88.4% sensitivity and 95.2% specificity (AUC = 0.93, p-value = 0.001) (Fig. 5d). ROC analysis indicated the diagnostic efficacy of both biomarkers in discriminating CD patients from controls.

### Logistic regression to measure the relationship between the serum biomarkers

**Fig. 6:**
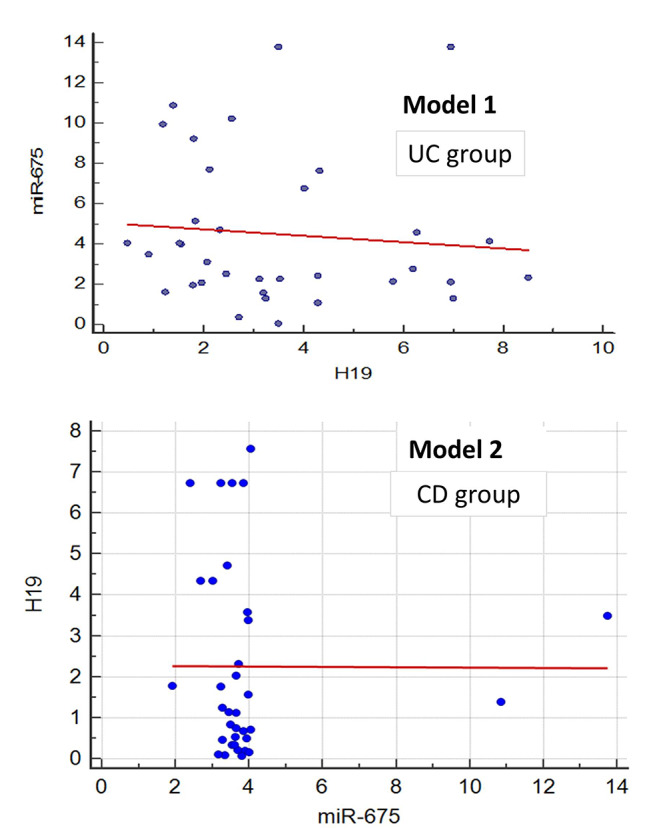
Logistic regression for the possibility of using miR-675-5p and H19 in predicting IBD. Model 1: Relationship between serum biomarkers for UC patient groups (miR-675-5p is the dependent variable). Model 2: Relationship between serum biomarkers for CD patient groups (miR-675-5p is the dependent variable)

For Linear regression, models were conducted to measure the relationship between the serum biomarkers. We discovered a statistically significant distinction between the examined cases. (Fig. 6). Model 1 for UC patients (miR-675-5p is the dependent variable): results showed a relationship between miR-675-5p and H19, where p-value = 0.001, the unstandardized coefficient is -0.157, and the standardized coefficient is -0.092. Model 2 for CD patients (miR-675-5p is the dependent variable): results showed a relationship between miR-675-5p and H19, where p-value < 0.0001, the unstandardized coefficient was − 0.043, and the standardized coefficient was − 0.266.

## Discussion

As a group of inflammatory, chronic, and remitting disorders of the GIT, IBD is linked to influential morbidity and mortality that results in a substantial burden to both the patient and the finances of the health care system [28].

Our results revealed that the estimated serum levels for H19 and miRNA-675-5p in the UC group in comparison to the control group have a high statistical difference with high sensitivity and specificity. The same results apply to the CD group. Besides, both biomarkers could discriminate between UC patients in remission and those with moderate activity.

In contrast to cancer biology, the role of H19 in IBD and other inflammatory or autoimmune diseases has been scarcely studied [7]. H19 was dramatically upregulated in inflamed colonic tissues in patients with IBD [23]. Also, miR-675-5p was significantly upregulated in UC colonic mucosal biopsies [29]. In a recent study, H19 and miR-200a showed diagnostic significance in IBD patients [30].

H19 may be linked to intestinal inflammatory responses due to its effect on decreasing vitamin D receptor (VDR) expression in colonic biopsies in UC, which was partly due to miR-675–5p, the latter targets the 3′- untranslated region of VDR mRNA [31]. Over and above, H19 overexpression, associated with increased abundance of miR-675p- enhances intestinal permeability and decreases the expression of tight junction proteins and adherens junction, a matter that directly destabilizes the sturdiness of the intestinal mucosal barrier [25]. Recently, Yin et al. [32] found that silencing H19 could attenuate intestinal injury in UC mice.

In this study, CD patients tend to have more extra-intestinal manifestations than UC patients. A finding that comes in agreement with Isene et al. [33]. Also, we found significantly increased levels of CRP and ESR in the UC and CD groups compared to the control group, a significant elevation in total leucocyte count (TLC), neutrophils, and platelets, and decreased albumin levels. Although these laboratory investigations showed significance, they are not specific enough to rely on to diagnose IBD, so we need more specific and sensitive noninvasive biomarkers to rely on. A new study [34] found that serum levels of H19 and CRP are consistently associated with the clinical diagnosis of UC, with a superior conformance of H19 to that of CRP. The combination of different parameters showed higher accuracy than single marker approaches.

## Conclusion

In this study, we concluded that lncRNA H19 and its precursor miR-675-5p are overexpressed in inflammatory bowel disease, promising to be used as noninvasive, reliable biomarkers for IBD diagnosis.

## Data Availability

This manuscript contains all of the data produced during this research.

## Abbreviations

lncRNA: Long noncoding RNA
miRNAs: microRNAs
IBD: Inflammatory bowel disease
UC: Ulcerative colitis
CD: Crohn’s disease
GIT: Gastrointestinal tract
CRP: C-reactive protein
ZO-1: Zonula occludin 1
CBC: Complete blood count
ESR: Erythrocyte sedimentation rate
CDAI score: Crohn’s disease activity index
VDR: Vitamin D receptor
